# Construction Notes

**Published:** 1907-03-09

**Authors:** 


### CONSTRUCTION NOTES.

### TONBRIDGE COTTAGE HOSPITAL.

The enlargement to this hospital, recently completed, has now been opened. The hospital itself was opened in 1902, and the extension is practically an entire renovation of the institution. It comprises two new wards, with their usual sanitary annexa, built at a cost of over £1,780.

### CHESTER GENERAL INFIRMARY.

At the last annual meeting the Governors authorised the erection of a new laundry; this has now been completed and is proving of great value to the institution. Through the generosity of Mrs. Richard Tidswell, the operating theatre has been entirely renovated and refurnished at a cost of £620. The mortuary, which was previously in a very unsatisfactory state, has also been altered and renovated, the expense having been met by Miss Addis and the members of the nursing staff, who together collected more than £100 for that purpose. The Nurses' Home has been repainted and restored.

### GRIMSBY HOSPITAL.

During the past year important improvements have been made in this hospital. A new lift has been erected, through the initiative of Mr. C. F. Carter, and the kindness of many friends of the hospital, and is now in working order, and a great boon to the patients. No. 74 Yarborough Street has been bought for a porter's house, so that the porter is always within call. A new steriliser and rheostat have been purchased, with most satisfactory results. The fire appliances and drainage have been thoroughly overhauled, and the fire escape properly railed off. The night nurses' bedrooms, which were exceedingly damp, have been improved. Finally the "John Wintringham" Convalescent Ward has been prepared for reception of patients in case of great emergency.

### DANVERS HOSPITAL, U.S.A.

Two buildings have been erected of capacity sufficient to accommodate all the tuberculous patients in the hospital. Each building shelters fifteen patients. They are practically alike, one for women and the other for men. They are about 76 feet long and 21 feet wide. They front the south. Each has a piazza 10 feet wide running the whole length of the front of the building. The front is nearly all glass, and there is considerable glass in other portions of the walls. The windows are double, without guards, and above each window is a glass transom, and all can be raised or swung. The buildings are heated by steam pipes running beneath the windows on three sides of the wards. Fresh air is taken directly through the walls. A sheet-iron arrangement causes the air to flow over the steam pipes before it enters the wards. Each building has two wards, 30 feet by 20 feet, 13 feet high, with a room between them 16 feet by 20 feet, containing a fireplace.

### WOMEN AND CHILDREN'S HOSPITAL.

Miss Standering, of Audus Street, Selby, has made arrangements to build, equip, and endow a hospital for the use of women and children of the township. There will also be added a much-needed operating theatre and other buildings for the joint use of the patients of Brooke Dispensary and the Cottage Hospital. The institution, which will be attached to the Brooke Dispensary and Cottage Hospital, will be styled "The Standering Memorial Ward," in memory of the donor's late brother and sister, and will be managed by the Governors of the Brooke Dispensary and Cottage Hospital in conjunction with two trustees nominated by the donor. The institution will be endowed with £3,000, exclusive of the cost of its site, purchased from the Earl of Londesborough; £2,000 will be provided at once, and the balance at the donor's death. The donor, with Colonel Hawdon, Mr. Mark Scott, and Mr. W. H. Staniland, are constituted the first trustees of the endowment, and the work of the erection of the buildings will commence at once.

### LYING-IN HOSPITAL, CITY ROAD, E.C.

After the annual meeting last week an opportunity for an inspection of the new building was afforded, under the guidance of Mr. M. E. Collins, the architect. The whole building has been planned with a view to render it as aseptic as possible, and to this end all the fittings and smallest details of construction have been completed with the most elaborate care. The hospital consists of a basement: engine-room, porters' room, etc.; ground floor: receiving department, dispensary, kitchens, board-room, and medical superintendent's suite; the first and second floor, containing wards, delivery rooms, matron's rooms; with an isolation ward, which is reached by the roof, and is distinct from the rest of the building. In order to ensure aseptic conditions, the women who apply for admission enter by a side door into a room fitted with glass wall-linings and terrazzo jointless floor, which can be cleansed with hose-pipe in every part. After they have been interviewed by the assistant secretary, who only speaks to them through a window, thus avoiding all direct contact, they pass out again into the street without entering the main building. When the patients enter the hospital it is by another door, and their clothes having been removed, they are washed in an adjoining room before going upstairs. Near by there is a linen drying-room, work-room, and storing-room, which have not only jointless flooring, but also "fram" ceilings, to prevent the accumulation of any dust or germs. Throughout the hospital the wood fittings are all rounded; all pipes, basins, baths, radiators, are fixed free from the wall; the ceilings are all absolutely flat, but rounding on to the walls, the doors of the wards are made of one solid piece of wood, there are no beams throughout, every window is flush, and it is indeed hard to enumerate the multifarious ways in which Mr. Collins has put into practice his principle of aseptic construction.

There are nine large and two small wards. Each ward is absolutely apart from the rest of the building, having what may be termed a small ante-room with through current of air, and all the ventilation is independent, so that isolation would be easy if required. Accommodation is provided for about 60 beds. Of the two delivery-rooms, one is especially fitted with a top-light and external heating, and in both there is a washing apparatus that can be turned on and off by the foot and elbow to facilitate cleansing after operating, and fresh air radiators of a patent kind. In addition, there is a room specially designed for a patient in collapse where an unobstructed current of air may be secured in a moment. There is also a babies' room containing eight basins with special water apparatus and drying pipes around the walls. All soiled linen is thrown down a caging through a window and either burnt in special cinerators or disinfected and washed before being sent to the laundry. There are two fire-escapes, and all electric lights are separately laid on.

